# Precision epitope editing: A path to advanced immunotherapies

**DOI:** 10.1016/j.cellin.2024.100226

**Published:** 2024-12-24

**Authors:** Rui-Jin Ji, Mu-Yao Wang, Ying Zhang

**Affiliations:** a Esophagus, Mediastinum and Lymphatic Oncology Department, Medical Research Institute, Frontier Science Center for Immunology and Metabolism, Zhongnan Hospital of Wuhan University, Wuhan University, Wuhan, 430071, Hubei, China; b Department of Rheumatology and Immunology, Medical Research Institute, Frontier Science Center for Immunology and Metabolism, Zhongnan Hospital of Wuhan University, Wuhan University, Wuhan 430071, Hubei, China; c TaiKang Centre for Life and Medical Sciences, TaiKang Medical School, Wuhan University, Wuhan, 430071, Hubei, China; d State Key Laboratory of Virology, Wuhan University, Wuhan, 430071, Hubei, China

**Keywords:** CRISPR, Epitope, Prime editing, Precision editing, HSPC, AML

## Abstract

The ability to recognize antigen epitope is crucial for generating an effective immune response. By engineering these epitopes, researchers can reduce on-target/off-tumor toxicity associated with targeted immunotherapy. Recent studies indicate that employing various gene editing tools to modify the epitopes of healthy hematopoietic stem and progenitor cells (HSPCs) can protect these cells from toxicity during tumor eradication, all while preserving their differentiation and function. This advancement greatly enhances the safety and efficacy of tumor immunotherapy.

## 1 Introduction

**Fig. 1:**
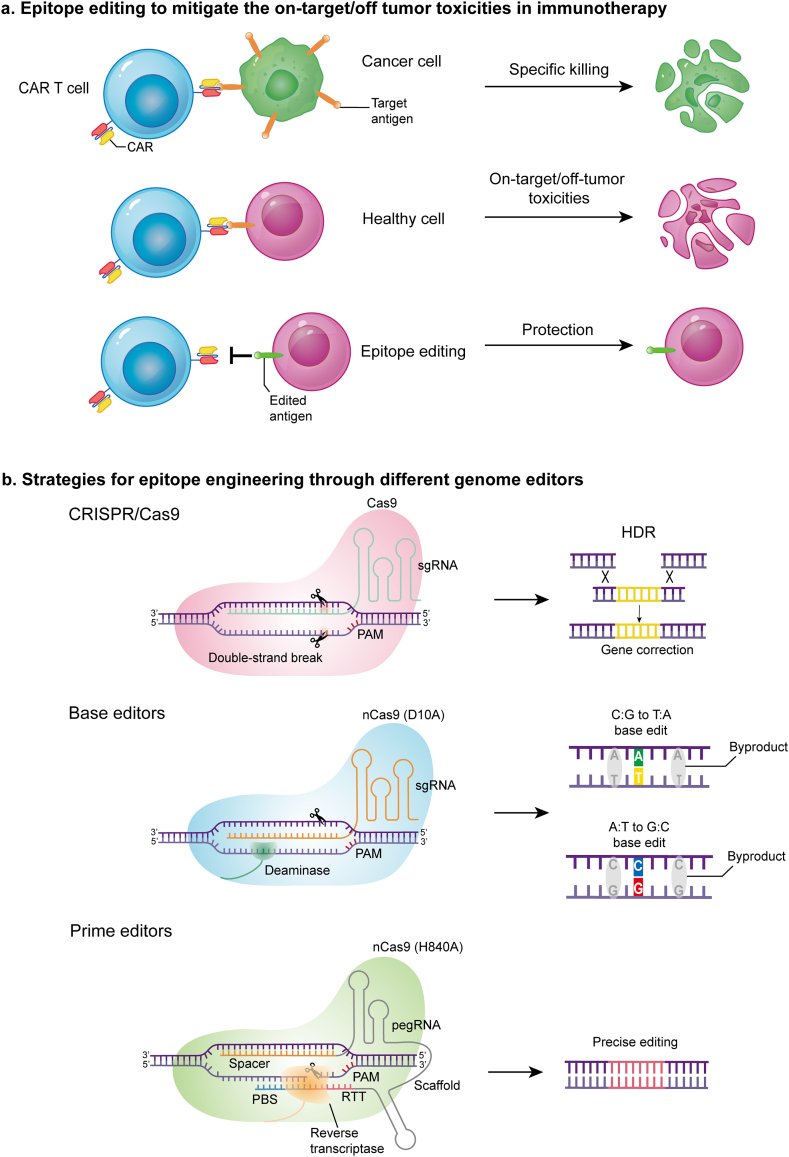
**Strategies for antigen epitope engineering** a. Epitope editing to mitigate the on-target/off tumor toxicities in immunotherapy. b. Strategies for epitope engineering through different genome editors, such as CRISPR/Cas9, base editors and prime editors to modify antigen epitope. PAM, protospacer adjacent motif; HDR, homology-directed repair; nCas9, Cas9 nickase.

Over the past decade, immunotherapies have revolutionized cancer treatment by harnessing the nature of immune system to fight against pathogenic cells (Hiam-Galvez et al., 2021). Through modulating patients' immune responses with immune checkpoint inhibitors, such as anti-CTLA-4, anti-PD1, and anti-PD-L1, a variety of tumor types can be effectively treated (Kubli et al., 2021; Liu et al., 2024; Sharma et al., 2023; Zhang et al., 2024). These treatments have shown profound effects in conditions like melanoma and non-small cell lung cancer (Champiat et al., 2016; WANG, KIM, et al., 2022). Despite the success, these therapies benefit only a subset of patients and can lead to immune-related adverse events (Morad et al., 2021). In addition to checkpoint inhibitors, therapies such as chimeric antigen receptor (CAR) T cells and antibody-drug conjugates (ADC) are gaining traction in the treatment of hematological malignancies (An et al., 2024; LABANIEH & MACKALL, 2023; Tsuchikama et al., 2024), and have shown promise in solid tumors (Choi et al., 2024; Hegde et al., 2020; Watanabe et al., 2018). CAR-T therapy targeting CD19 has demonstrated remarkable success in treating B-cell acute lymphoblastic leukemia (Grupp et al., 2013; Ying et al., 2019). Additionally, CAR-T and monoclonal therapies targeting antigens such as CD269, CD123, and CD7 are being investigated in the immunotherapy of other hematological malignancies (Saez-Ibañez et al., 2022; Savani et al., 2021). Despite these advances, the heterogeneity and complexity of antigen expression in tumor cells often complicate treatment. Tumor-specific antigens are often expressed in non-malignant cells, and CAR-T immunotherapy can lead to healthy cells eradiation. This on-target/off-tumor toxicity causes severe side effects and, in some cases, even death (Hofmann et al., 2019; Shimabukuro-Vornhagen et al., 2022). This review summarizes recent studies where researchers have used genome editing tools to modify antigenic epitopes in healthy cells (Fig. 1). These modifications aim to mitigate the adverse effects of on-target toxicity while preserving the normal function of antigen in healthy cells.

## 2 Brief introduction of genome editing tools

Since its discovery in 2012, the CRISPR/Cas (Clustered Regularly Interspaced Palindromic Repeats/CRISPR-associated genes) system has revolutionized the life sciences owing to its simplicity and efficiency (Gasiunas et al., 2012; Jinek et al., 2012; Wu et al., 2023; Yang et al., 2022; Zhang, Zhang, et al., 2023). This powerful genome editing technology has also demonstrated significant potential in disease treatment as evidenced by the approval of first gene editing drug CASGEVY in 2023 (Kliegman et al., 2024; Wang, Gao, et al., 2022). CRISPR/Cas9 employs a single guide RNA (sgRNA) to direct Cas9 nuclease to the target sequence which shares complimentary sequence to sgRNA. Cas9 then cleaves the target and non-target DNA strands through its HNH and RuvC nuclease domains, resulting in double-strand breaks (DSBs) at the target loci (Price & D'Andrea, 2013; Jiang, Doudna, 2017; Shi et al., 2022). DSBs are primarily repaired through two pathways: non-homologous end joining (NHEJ) and homology-directed recombination (HDR) (Nambiar et al., 2022). NHEJ leads to small insertions or deletions (indels) at the break site, while HDR precisely makes desired edits at the break site if presented with a DNA repair template (Zhang, 2021). Studies have indicated that DSBs created by SpCas9 can lead to large-scale deletions of DNA segments and activate the p53 pathway, potentially resulting in genome instability and cell cycle arrest (Haapaniemi et al., 2018; Ihry et al., 2018). Furthermore, the efficiency of HDR is limited in non-cycling or slowly cycling cells due to its dependence on the cell cycle, strongly restricting its therapeutic application as most therapeutic relevant cell types are slow-cycling in nature (Heyer et al., 2010; Paquet et al., 2016; Qiu et al., 2022).

To overcome the limitations, researchers have developed base editors by fusing either cytosine deaminase or an engineered adenine deaminase to a nicked Cas9 nuclease (D10A). There are two primary types of base editors: adenine base editors (ABEs) and cytosine base editors (CBEs), which convert adenine (A) to guanine (G), or cytosine (C) to thymine (T), respectively (Anzalone et al., 2020; Komor et al., 2017). Base editors can correct certain point mutations without generating double-strand breaks (Gaudelli et al., 2017; Komor et al., 2016). Despite their robust on-target activity, base editors are susceptible to bystander editing, wherein neighboring nucleotides within the editing window are converted, resulting in unintended edits (Grünewald et al., 2019). Protein engineering of base editors can reduce bystander editing by either narrowing the editing window (Chen et al., 2023; Gehrke et al., 2018; Wang et al., 2018) or restricting editing to deaminases with a more specific motif preference (Gehrke et al., 2018; Liu et al., 2020; Wang et al., 2018). Because the deaminase catalyzes reactions on single-stranded DNA (ssDNA) or RNA substrates, the presence of dynamic ssDNA due to transcription or DNA replication in cells could lead to substantial off-target editing activities (Jin et al., 2019; Zuo et al., 2019). Considerable efforts have been made to reduce the off-target activities. For example, the transformer base editor (tBE) employs the cleavable deoxycytidine deaminase inhibitor (dCDI) domain, which is connected to the cytidine deaminase. When tBE binds to the target site, a truncated helper sgRNA (hsgRNA) positions the protease near the on-target site to cleave the deaminase inhibitor, allowing targeted editing. This mechanism keeps the tBE inert and reduces the generation of off-target mutations (Han et al., 2023; Wang et al., 2021).

In response to challenges associated with base editors, prime editor (PE) was developed to enable all twelve nucleotide conversions with high precision (Anzalone et al., 2019). Prime editor is composed of a Cas9 nickase (H840A) fused with reverse transcriptase (RT) and a prime editing guide RNA (pegRNA). The pegRNA contains a classic sgRNA fused with a primer binding site (PBS), and an RT template (RTT) fused into one RNA (Anzalone et al., 2019). When bound to the target sequence, nCas9 nicks the non-targeting DNA strand, allowing the PBS to hybridize with the exposed DNA flap and enabling reverse transcription using the RTT as a template. This generates a 3′ DNA flap that integrates seamlessly into the target site (Anzalone et al., 2019). PE has been shown to successfully make precise small insertions and deletions in multiple cell types with high precision and little off-target activities (Gao et al., 2022; Zhu et al., 2024). The nature of cell cycle independence has made PE particular attractive for therapeutic applications. While many improved PE variants have been developed (Anzalone et al., 2020; Chen, Liu, 2023), the efficiency of PE in primary cells vary significantly across genomic loci (Yan et al., 2020; Yang et al., 2019). Whether PE can be employed to treat disease remains unclear (Everette et al., 2023; Sousa et al., 2024).

## 3 Engineered targeted immunotherapies

CAR-T therapy has been successful in treating B-cell malignancies. This is because CAR-T induced on-target/off-tumor toxicities of B-cell aplasia in patients can be managed with regular immunoglobulin infusions (Porter et al., 2011; Sheykhhasan et al., 2022). However, in other hematologic malignancies, such as acute myeloid leukemia (AML), the primary obstacles to successful CAR-T immunotherapy have been the on-target/off-tumor toxicities, which often lead to clinical setbacks. AML is characterized by the abnormal differentiation of hematopoietic stem and progenitor cells (HSPCs) in the bone marrow and blood (Döhner et al., 2015). Chemotherapy and allogeneic hematopoietic stem cell transplantation (allo-HSCT) are the standard treatments for AML. Because of the high relapse rate, the five-year survival rate of AML remains low (Jongen-Lavrencic et al., 2018). Due to the shared expression of molecular targets on both cancerous and healthy hematopoietic progenitor cells, CAR-T immunotherapy against AML faces severe on-target/off-tumor toxicity of eradicating healthy hematopoietic progenitor cells (Atilla & Benabdellah, 2023; Flugel et al., 2023; Wang et al., 2015). Building on advancements in genome editing, researchers are developing innovative strategies to mitigate toxicity and enhance the safety of AML treatments (Fig. 1).

The initial efforts of engineered targeted immunotherapy focused on the CD33 antigen, a member of the sialic acid-binding immunoglobulin-like lectin (Siglec) family, which has been shown to be dispensable for normal hematopoiesis in mouse models (Brinkman-Van Der Linden et al., 2003). Kim et al. employed the CRISPR/Cas9 system to knock out CD33 in HSPCs, resulting in resistance to CD33-targeted CAR-T therapies while preserving normal hematopoietic function in mouse. Furthermore, they demonstrated that CD33 knockout HSPCs have the potential for long-term multilineage engraftment in non-human primates (Kim et al., 2018). In a separate study, Borot et al. demonstrated that CD33-ablated HSPCs could be combined with CD33-targeted immunotherapies, such as CAR-T and monoclonal ADC Gemtuzumab Ozogamicin (GO) (Borot et al., 2019). Another approach by Humbert et al. targeted the exon 2 of CD33 to remove the V-set immunoglobulin-like domain critical to existing CD33-directed therapies, resulting in the expression of a shorter isoform, CD33^ΔE2^, which may reduce adverse effects associated with complete CD33 expression loss (Humbert et al., 2019). In addition, a base-editing screen targeting all canonical splice donor and acceptor sites of CD33, identified several single-base edits that could reduce or eliminate CD33 expression in HSPCs (Martin-Rufino et al., 2023). While studies have demonstrated the feasibility of knocking out CD33, with promising results showing a favorable safety profile and successful engraftment of CD33-deficient HSPCs, the long-term effects of CD33 knockout on hematopoietic function remain unclear. With the initiation of clinical trials evaluating this CD33 knockout strategy (NCT04849910 and NCT05945849), human data are anticipated to clarify these concerns.

Targeting a dispensable antigen carries the risk of immune escape, as tumor cells may downregulate antigen expression to survive (Majzner, Mackall, 2018). In addition, the knockout approach is limited to antigens that are not essential for hematopoietic function. To address these concerns, we and others hypothesize that introducing a function-preserving mutation within the epitope of the target antigen using genome editing tools could maintain antigen functionality while minimizing on-target/off-tumor toxicity.

CD123 is a canonical AML biomarker (Testa et al., 2014), whose function is crucial for mediating interleukin-3 (IL-3) signaling across various cell types (Dougan et al., 2019; Yi et al., 2022). To precisely modify the CD123 epitope against clinically validated anti-CD123 scFv, CSL362 (Broughton et al., 2014), three research groups employed different editing strategies to alter the epitope (Casirati et al., 2023; Ji et al., 2024; Marone et al., 2023). Casirati et al. performed a base-editing screen, and identified CD123 S59P as an optimal mutation that resisted CAR-T cell binding. CD123 S59P-edited HSPCs, transplanted into immunodeficient mice, demonstrated successful engraftment, lineage differentiation, and stable edited cell populations while reducing CAR-T cell toxicity. To confirm dual-edited HSPC resistance to combined CAR-T cell therapy, they simultaneously transplanted dual FMS-like tyrosine kinase 3 (FLT3) N399/CD123 S59-edited into mice models. Given the high heterogeneity of AML, single-target CAR-T therapies may lack efficacy in fully eradicating tumors. The potential of multiplex editing in HSPCs paves a way for dual CAR-T treatments with reduced off-tumor hematopoietic toxicities. Different than aforementioned work, Marone et al. chose the E51K epitope as it has minimal effect on CD123 function whereas the S59P epitope selected by Casirati work, could impact the ligand interaction to CD123 (Marone et al., 2023). To prove the concept, they used CRISPR/Cas9-mediated HDR to engineer the E51K epitope of CD123 in HSPCs, showing epitope edited-cells are protected from CAR-T killing.

While HDR-based precision editing has low efficiency in quiescent long-term repopulating HSCs (LT-HSCs) and base editors pose the risk of bystander editing (Ferrari et al., 2020; Genovese et al., 2014; Dudek et al., 2024; Wilkinson et al., 2021; Schiroli et al., 2019), the precision of prime editors offers a promising alternative for epitope editing. In our recent study, we employed both base editors and a prime editor to modify the epitope of CD123 on HSPCs (Ji et al., 2024). To identify CD123 variants that evade recognition by the clinically tested scFv-CSL362, our group developed a binding affinity assay and conducted three rounds of screening focused on the CD123 N-terminal domain (NTD) surface. We selected the E51K and R84Q variants for their significantly reduced antibody binding affinities while retaining normal protein expression and post-translation modifications (PTM). The previously identified CD123 S59P variant also showed resistance to scFv binding in our screen, but detailed analysis indicated that S59P variant led to altered PTM. PTMs, such as glycosylation, can shield glycoproteins from proteolytic digestion due to limited accessibility of sugar chains. Altered glycosylation can reduce protein stability, impacting expression and function in hematopoietic cells. Thus, rigorous assessment of surface protein modifications is essential, as this factor has often been overlooked in previous research.

When using base editors to modify the epitope of R84Q and E51K, the bystander products of E51K led to complete protein expression loss. Though the majority of R84Q bystander products have less impact on protein expression, the complex products remain a concern. To improve the precision, we employed prime editors and selected the double mutations of R84Q-V85M for further validations, as this dual variant exhibited 10-fold lower binding affinity than the R84Q variant. To improve the editing efficiency, we conducted several rounds of optimizations including codon-optimization of RTT, ligated chemically modified epegRNA, double electroporation delivery and mRNA formulation (An et al., 2024; Lei et al., 2024; Wang, Cheng, et al., 2022; Zhang, Qiu, et al., 2023). These efforts finally raised the epitope editing efficiency in HSPCs from 5.9% to 78.9%, with a purity of 76.3%. PE-modified HSPCs were resistant to CAR-T cell-mediated lysis in both cell and immunodeficient mice models, underscoring the precision and robustness of prime editing for epitope modification and providing a proof-of-concept strategy for precision epitope engineering (Yin, Li, 2024).

CD45 is a pan-leukocyte marker expressed on most hematopoietic cells, including those in hematologic malignancies (Hermiston et al., 2003). As a receptor tyrosine phosphatase, CD45 plays a critical role in regulating immune response and cellular homeostasis (Mujal, Krummel, 2019). Loss of CD45 leads to severe combined immunodeficiency (Kung et al., 2000). Because of the essential function of CD45, two research groups employed base-editor epitope engineering and demonstrated that epitope-modified HSPCs are protected from CAR-T cell-mediated eradication (Garaudé et al., 2024; Wellhausen et al., 2023). These studies highlight the potential of this approach to deliver a universally applicable and less toxic immunotherapy for various hematologic malignancies.

Collectively, these studies illustrate the progress from antigen knockout approaches to precision epitope engineering, greatly improving the precision and effectiveness of immunotherapies for blood cancers. However, several limitations still need to be addressed. Epitope modifications might reduce the binding affinity of CAR-T cells or ADCs to the edited antigen, while they may not entirely eliminate binding. This residual affinity can result in unintended targeting and subsequent elimination of the engineered cells. Furthermore, alteration in the epitope may elicit an immune response against the new antigen. Even a single amino acid change carries the risk of creating an immunogenic epitope, potentially leading to the destruction of engineered cells by the recipient's immune system. Predicting whether an epitope modification will provoke the immune response is challenging due to the variability in antigen processing and presentation across different human leukocyte antigen (HLA) haplotypes. Immunosuppressive treatments used in allo-HSCT could help minimize the response to the engineered proteins; however, the long-term effects still remain unknown. The data presented indicate that epitope editing does not impair hematopoietic cell function, though further evaluation in an immunocompetent model is needed to confirm whether this modification affects protein function. Despite the overall safety observed in these studies, potential off-target effects from gene editing tools should be carefully considered. Off-target effects may lead to unintended genetic changes, disrupting normal cellular functions or causing immune responses that could undermine the safety and efficacy of a therapy. These risks highlight the importance of developing more precise editing tools and robust detection methods to faithfully capture such effects. As with any monotherapy, tumor relapse or drug resistance remains a concern. These adaptations could render the therapy less effective over time, underscoring the need for continuous monitoring and potential combination strategies to maintain therapeutic efficacy. If antigen escape or drug resistance occurs, donor lymphocyte infusions may be necessary for consolidation. Alternatively, multi-target immunotherapy using multiplex shielded HSPCs could help address resistance. Comprehensive preclinical and clinical studies are essential to understand and mitigate these risks, ensuring that epitope editing achieves its full potential as a safe and effective therapeutic strategy. While PE generally exhibits low efficiency in primary cells, our study highlights effective optimization of PE for epitope editing. However, additional clinical evidence is needed to confirm whether PE-edited HSPCs can effectively reconstitute the hematopoietic system and achieve the required efficiency for curative therapies in humans.

In summary, epitope editing enhances immunotherapy by increasing the precision of cancer-targeted treatments in clinical practice. By modifying the epitope of HSPCs, it offers a safer and more effective solution, particularly in cases where on-target/off-tumor toxicities present challenges. This strategy also offers potential for other applications, including *in vivo* selection of genome-edited HSCs without genotoxic conditioning, treatment of autoimmune diseases (Mackensen et al., 2022; Wang et al., 2024), and even eradication of HIV infection. This innovative approach not only facilitates cancer-specific therapies while preserving hematopoietic function but also paves a way for treating a wider range of diseases.

## CRediT authorship contribution statement

**Rui-Jin Ji:** Writing – original draft. **Mu-Yao Wang:** Writing – original draft. **Ying Zhang:** Writing – review & editing.

## Declaration of competing interest

Given her role as the Associate Editor, Y.Z. had no involvement in the peer-review of this article and has no access to information regarding its peer-review. The rest authors declare no competing interests.
